# Medical Students Understanding of The Scope of Plastic Surgery

**DOI:** 10.1055/a-2219-2411

**Published:** 2024-01-24

**Authors:** Mohammad K.H.B. Abdulaziz, Mohammad Al-Jamali, Sundus Al-Mazidi, Sarah Albuloushi, Ahmad B. Al-Ali

**Affiliations:** 1 Department of Plastic Surgery, University Hospitals Birmingham NHS Foundation Trust, Birmingham, Warwickshire, United Kingdom; 2 Department of Plastic Surgery, University Hospital Coventry, Coventry, United Kingdom; 3 Department of Plastic Surgery, Al-Babtain Center for Plastic and Burn Surgery, Kuwait City, Kuwait; 4 Department of Psychiatry, University Hospitals Birmingham NHS Foundation Trust, Birmingham, Birmingham, United Kingdom; 5 Department of Opthalmology, Al-Bahar Center for Opthalmology, Kuwait City, Kuwait

**Keywords:** plastic surgery, medical students, general surgery, ophthalmology, orthopaedic, ENT, neurosurgery, maxillofacial surgery

## Abstract

**Background** Plastic surgery has developed to benefit in a variety of challenging areas formerly handled by other disciplines. Medical students do not have a clear picture of plastic surgery as a career due to lacking scope, clinical practice, and understanding of plastic surgery as a clinical area of expertise, including general practitioners, nursing staff, medical trainees, and the general public, and misconceptions about the extent of reconstructive and plastic surgery.

**Methods** A cross-sectional observational study was conducted on Kuwait University Medical students (2nd–7th Years) over a period of 1 month. A questionnaire and a consent form were provided to eligible students. The inclusion criteria were Kuwait University Medical students from 2nd to 7th Years with signed consent form. The response was collected via email sent in coordination with the Vice Dean of Student Affairs in the Faculty of Medicine. Using statistical package for the social sciences, responses were statistically analyzed. Pearson's chi-square test was used to calculate *p* -values, where *p* < 0.05 was considered statistically significant.

**Results** A total of 244 eligible medical students, 121 males and 123 females, were included in the study, with a mean age of 21 (±2) years. Similarly, 126 (51.6%) were preclinical students (2nd–4th-year students), while 118 (48.4%) were clinical students (5th–7th-year students). About 79.8% of medical students believed that plastic surgery plays an essential role in trauma management, whereas 9.2% did not consider plastic surgery significant for trauma management. This study found that only 15.5% of medical students were interested in enrolling in plastic surgery residency after graduation, while 47.1% of students did not consider plastic surgery residency after graduation. However, 37.4% were uncertain. The two most driving factors in deciding on plastic surgery residency were expected income (61.8%) and lifestyle (14.3%).

**Conclusion** Improving medical students' education quality can enhance their perception and awareness of plastic surgery. Students should be taught the broader scope of plastic surgery. The inclusion of formal training during undergraduation is the essence of time and should be added to or improved during plastic surgery rotations with more emphasis on reconstructive and hand/peripheral nerve surgery. Student-led interest groups can be a useful tool for educating students about their specialty.

## Introduction

Plastic surgery is a surgical discipline focused on reconstructing facial and body tissue defects. The imaginative aspect of plastic surgery generates a distinctive atmosphere in which the surgeon's creativity is the sole constraint. The surgeon's creativity leaves individuals outside the area perplexed about what a plastic surgeon accomplishes. There are several dimensions to cosmetic procedures—a progressive and developing field not constrained by anatomical or organ systems. Not limited to breast treatments, maxillofacial trauma, cleft lip procedures, skin cancer, burns, hand treatments, trauma reconstruction, cosmetic operations, oncology transformation, ophthalmic eye procedures, etc., are all subspecialties of cosmetic procedures. 1 Plastic surgery has developed to benefit in a variety of challenging areas formerly handled by other disciplines. 2 In addition, general practitioners, nursing staff, medical trainees, and the general public do not entirely understand plastic surgery as a clinical practice. 3 The general public and medical experts have misconceptions about the extent of reconstructive and plastic surgery. 4

Studies suggest that financial, professional possibilities, social status, ambitions, personal traits, location advantages, and lifestyle benefits are among the most important factors influencing medical students' decision to pursue a certain discipline. In addition, in Saudi Arabia, lifestyle offered by any specialization has been the most important element affecting medical students' decisions about their medical specialties. 5 A study showed that in Saudi Arabia, boys and girls tend to favor general surgery and dermatology, respectively, in medicine. 6 Numerous studies have observed the variables that might affect a person's decision to pursue a career in plastic surgery globally. Very often, plastic surgery is considered a cosmetic treatment in the public. Major misunderstandings about plastic surgery result from the public's point of view, ultimately affecting medical students' perception with no access to this field during their clinical rotations. As a result, some medical schools have included clinical rotations for plastic surgery in their medical curricula to dispel this myth. 7

According to a survey, the predominant association between cosmetic procedures and financial benefits was a common reason for medical students' unfavorable perception of plastic surgery. 8 These negative perceptions severely affect students enrolling in plastic surgery as a career and professional referral practice. A survey shows that only 11% of medical graduates at prestigious institutions in the United Kingdom considered plastic surgeons were most inclined to conduct tendon surgery. 8 These medical students will eventually become professional doctors; thus, their preconceptions regarding plastic surgery might impact the field as a whole, altering referral trends, opinions, and if medical students decide to follow this as a career choice. 3 In a large, comprehensive examination of 122 full-text source publications, three key concepts—gender, surgical training characteristics, and student “fit” in the surgery environment—were found to influence operational career choice. 9

Literature reveals that several medical school courses undervalue plastic surgery, a debatable topic. Lack of proper training and career counselling in plastic surgery at the undergraduate level is a primary concern when medical students decide to choose plastic surgery as a specialty. As cosmetic surgery and other areas of expertise like maxillofacial surgery, orthopaedic procedures, dermatology, and otolaryngology frequently share procedures, those who backed the involvement of cosmetic procedures in medical programs claimed that this integration would enhance the referral trend among medical providers. 10 Additionally, exposing undergraduate medical students to plastic surgery will enhance their decision rates to specialize in this field. According to a survey, 30% of plastic surgery residents chose their field while attending medical school. 11 As a result, how medical students view plastic surgery may be important in determining whether they decide to pursue it as a profession after graduation. According to research, the majority of medical experts, teachers, and students agree that reduced exposure to plastic surgery curricula in medical school decreases students' professional proficiency as prospective surgeons, doctors, and regular practitioners. 12 However, the availability of knowledge and the aforementioned beneficial consequences strengthen the case for medical students' legitimate requirement of plastic surgery rotation regarding the impact of medical practice and education variations on medical graduates. The same regulations on medical procedures and specialization apply to medical students enrolled in various programs. Thus, the sort of medical education that graduate practitioners were exposed to might impact their inclinations for the profession and, later on, how they carry out health care choices and choose which patients to refer to plastic surgeons. 13

In Kuwait, without mentioning specializations other than primary health care, physicians were named as one of the resources and benchmarks for the growth of the health care workforce in the Ministry of Health yearly report for 2019. 13 As a result, accurate monitoring of national surgical employee training is lacking, which is crucial for strategic planning. Furthermore, the alarmingly low interest in surgery among medical students in Kuwait makes their medical careers at risk, necessitating an investigation of challenges and potential remedies, primarily since no prior local research has addressed this topic. 14 Over 800 students are now enrolled in the faculty's 7-year medical program at Kuwait University. The academic system is built on a problem-based teaching method, with the first 3 years being devoted to basic sciences and the latter 3 years being medically focused. Workshops and seminars on plastic surgery are 2 days long overall, focusing on the fundamentals. The perception of medical students at Kuwait University about plastic surgery is inadequate and does not sufficiently reflect the range of the profession. However, with appropriate practical experience, this may improve.

### Aim/Objective of Study

This study aimed to investigate Kuwaiti medical students' perception of plastic surgery in terms of influence, passion, and career prospective. Additionally, to evaluate the comprehension, competition, interest, and quality of education in plastic surgery and devise approaches to increase undergraduate interest in the field.

## Methods

### Study Setting

The study was conducted in Kuwait Medical University.

### Study Duration

The study was conducted over a period of 1 month.

### Inclusion Criteria

- Kuwait Medical University students
- From 2nd to 7th Years
- Those who signed the consent form

### Exclusion Criteria

- First-Year medical students of Kuwait University
- Medical students from other than Kuwait University
- Medical students from Kuwait University who did not sign the consent form

### Data Collection

Monkey Software formulated a questionnaire-based survey. Data of eligible participants based on inclusion criteria were collected over 1 month via university emails of the students in coordination with the Vice Dean of Student Affairs in the Faculty of Medicine. A total of 244 responses were collected, 121 males and 123 females. Similarly, students were categorized into preclinical (2nd, 3rd, and 4th Years) and clinical (5th, 6th, and 7th Years), respectively.

### Data Analysis

Data were analyzed using statistical package for the social sciences (SPSS) software (version 25; SPSS Inc, Chicago, IL). They were presented as continuous variables, that is, mean and standard deviation, and categorical variables were expressed as count ( *n* ) and percentage (%). Pearson's chi-square test was used to calculate *p* -values for categorical variables. *p* < 0.05 was considered statistically significant.

## Results

### Demographic Characteristics

A total of 244 students participated in the study, including 121 males and 123 females, respectively, with a mean age of 21 (±2) years. The number of students who participated in the survey from the 2nd, 3rd, 4th, 5th, 6th, and 7th academic years were 37 (15.2%), 42 (17.2%), 39 (16%), 42 (17.2%), 42 (17.2%), and 42 (17.2%) respectively ( Table 1 ). Similarly, categorically, 126 (51.6%) were clinical students (5th, 6th, and 7th Years), while 118 (48.4%) were preclinical students (2nd, 3rd, and 4th Years).

**Table 1:** Background characteristics and perception of plastic surgery subspeciality of study subjects by gender

Population background	Total ( *N* = 244)	Male ( *N* = 121)	Female ( *N* = 123)
	Number (%)		
Age (years, mean [±SD])	21 (±2)	21 (±2)	21 (±2)
Academic Year, ( *n* [%])			
7th	42 (17.2)	21 (17.4)	21 (17.1)
6th	42 (17.2)	21 (17.4)	21 (17.1)
5th	42 (17.2)	22 (18.2)	20 (16.3)
4th	39 (16)	19 (15.7)	20 (16.3)
3rd	42 (17.2)	20 (16.5)	22 (17.9)
2nd	37 (15.2)	18 (14.9)	19 (15.4)
Clinical academic year, ( *n* [%])			
Clinical students	126 (51.6)	64 (52.9)	62 (50.4)
Preclinical students	118 (48.4)	57 (47.1)	61 (49.6)
Plastic surgery perception, ( *n* [%])			
Do you think plastic surgery plays an essential role in trauma management?			
Yes	190 (79.8)	104 (87.4)	86 (72.3)
No	22 (9.2)	7 (5.9)	15 (12.6)
I don't know	26 (10.9)	8 (6.7)	18 (15.1)
Are you considering to apply to plastic surgery residency after graduation?			
Yes	37 (15.5)	13 (10.9)	24 (20.2)
No	112 (47.1)	62 (52.1)	50 (42)
Not decided yet	89 (37.4)	44 (37)	45 (37.8)
What are the two most influential factors that drive medical graduates to apply for plastic surgery residency?			
Variety of cases and operations	23 (9.7)	10 (8.4)	13 (10.9)
Positive personality traits	16 (6.7)	9 (7.6)	7 (5.9)
Expected income	147 (61.8)	84 (70.6)	63 (52.9)
Expected lifestyle	34 (14.3)	10 (8.4)	24 (20.2)
Media portrayal	18 (7.6)	6 (5)	12 (10.1)

Abbreviation: SD, standard deviation.

### Medical Students' Perception of Plastic Surgery

The study reported that 79.8% of medical students believed that plastic surgery is essential in trauma management, whereas 9.2% did not consider plastic surgery significant. A total of 112 (47.1%) students did not consider applying for plastic surgery residency after graduation. However, 89 (37.4%) were uncertain, and 37 (15.5%) were willing to apply to this field after graduation.

The two most influential factors reported by the medical graduates expected to drive them toward plastic surgery residency were expected income (61.8%) and lifestyle (14.3%). On the other hand, media portrayal (7.6%) and positive personality traits (6.7%) were reported as the least attractive factors ( Table 1 ).

### Responses of Clinical and Preclinical Medical Students Predicting the Most Suitable Specialty to Refer the Stimulated Cases

Plastic surgery was selected as the most preferred option by the medical students for clinical scenarios, including breast size reduction (96.4%), cosmetic nose reshaping (85.25%), cosmetic contouring and liposuction (84.84%), facial fillers' injections (65.57%), and abdominal chest burn experienced by firemen (58.26%). Despite the difference, there is no significant relationship between students' preference for plastic surgery and clinical scenarios; *X* ^2^ (396, *N* = 244) = 414, *p* > 0.05.

**Table:** 

Clinical scenarios and plastic surgery preference			
	Value	df	*p* -Value
Pearson's chi-square	414.000	396	0.257
Likelihood ratio	133.142	396	1.000

Medical conditions where students optioned plastic surgery infrequently (by <10% of the medical students) involved cases such as carpal tunnel syndrome (6.2%), fractured jaw (3.69%), skull deformity in a newborn (8.61%), severed ulnar nerve (9.43%), drooping eyelid affecting vision (6.56%), nasal septal deviation and breathing problems (8.61%), total hip replacement in elderly (4.51%), lacerations in hand tendon (9.02%), excision of a lipoma in the back (9.92%), brachial plexus injury due to motorcycle accident (4.13%), decompression surgery for protruding eye (4.55%), bowel obstruction (2.07%), cancer of the roof of the mouth (2.07%), and orbital fracture repair due to nail impinging the lower lid (5.37%).

Scenarios, where maxillofacial surgery was the most frequently chosen specialty include cleft palate syndrome (58.26%), fractured jaw (48.36%), and management of cancer in the roof of the mouth (43.38%). However, there was no statistical significance in clinical scenarios and choosing maxillofacial surgery as the speciality; *X* ^2^ (308, *N* = 244) = 322, *p* > 0.05.

**Table:** 

Clinical scenarios and maxillofacial surgery preferences			
	Value	df	*p* -Value
Pearson's chi-square	322.000	308	0.280
Likelihood ratio	104.683	308	1.000
Number of valid cases	23	–	–

Students mentioned that neurosurgery was the most frequently chosen specialty in cases involving carpal tunnel syndrome and tingling (41.32%), skull deformation in a child (63.52%), severed ulnar nerve (57.38%), and brachial plexus injury (48.76%). Despite the difference in choosing neurosurgery speciality by students due to various clinical scenarios, the relationship was statistically insignificant; *X* ^2^ (286, *N* = 244) = 299, *p* > 0.05.

**Table:** 

Neurosurgery speciality			
	Value	df	*p* -Value
Pearson's chi-square	299.000	286	0.287
Likelihood ratio	111.641	286	1.000

ENT was identified as the most suitable specialty for the management of cases including broken nose (41.39%), nasal septal deviation (80.33%), and absence of an outer ear during birth (51.64%); however, the selection was statistically insignificant; *X* ^2^ (286, *N* = 244) = 299, *p* > 0.05.

**Table:** 

ENT			
	Value	df	*p* -Value
Pearson's chi-square	299.000	286	0.287
Likelihood ratio	114.275	286	1.000

On the other hand, students selected ophthalmology most frequently in scenarios that involved drooping eyelid impacting vision (61.48%), decompression surgery for protruding eyes (73.97%), and repair of the fractured orbital plate (50.83%). Orthopaedics was selected in clinical scenarios that included muscle coverage for exposed tibia after an accident (54.1%), total hip replacement (86.89%), hand tendon lacerations (55.33%), and orbital fracture repair (9.92%).

The results of the present survey reveal that plastic surgery was selected as the most preferred option by the medical students for clinical scenarios including breast size reduction, cosmetic nose reshaping, cosmetic contouring and liposuction facial fillers' injections, and abdominal chest burn experienced by firemen. Similarly, in another study breast asymmetry and rhinoplasty were the most frequently recognized “cosmetic” treatments of plastic surgery, with recognition rates of 96.4 and 91.2%, respectively. These results are consistent with past surveys of medical students indicating that there is still room for improvement in the way that medical students are taught about the range of plastic surgery. 8 15

## Discussion

Study findings report a lack of adequate knowledge regarding the clinical aspect of plastic surgery. Medical students from 2nd to 7th academic years were provided a clear view of career prospective in plastic surgery. Unexpectedly, despite a presumed level of clinical exposure in the field, neither the year of study nor gender was associated with a future interest in a career in plastic surgery. Alarmingly, during the past 10 years, interest in pursuing a career in surgery has significantly decreased overall. Supplementary Table 1 summarizes that medical students consider plastic surgery a profession not associated with trauma management and only mainly desirable for economical income; which is alarming finding. 16

Plastic surgery is acknowledged for its wide range of procedures as well as patient variability deals by plastic surgeons during day-to-day practice. 5 This particular aspect propels 53.1% of the population to choose plastic surgery as their future specialty. 5 Interestingly, 25% of the participants in the Pasha et al's study 17 rated it as the most alluring aspect. As Table 1 demonstrated, desirable expected income was a main determinant in choosing plastic surgery as future career in among 65.5% students. Similar results were supported by Greene and May, 18 and it was also a significant predictor of job satisfaction among a cohort of Saudi-based physicians, as reported by Aldrees et al. 19 Furthermore, as this study reports 112 (47.1%) students who did not consider to enrol in plastic surgery residency after graduation. Studies report several factors that could result a significant drop in adopting plastic surgery by medical students such as, but not limited to, experience of undergraduate students, work–life harmony, perceived competition, and a shortage of surgical mentors, and negative connotation via media. 8 However, more concerningly, medical students, implying that the specialty and associated treatments are not well covered in the medical curricula and medical care plans, 7 have also misunderstood plastic surgery.

Improving the quality of education of medical students, who stand-in for the future of medicine, is one method to enhance their perception and awareness about plastic surgery. A study conducted in University of Utah, about understanding of plastic surgery as a career, allowing the medical educators to pinpoint particular areas in the curriculum that require improvement. This approach can be used by different medical schools to evaluate their students' understanding and develop novel strategies to improve medical students' knowledge of plastic surgery. 15

In this study, however, only 29.52% mentioned plastic surgery as primary surgery. Medical students' knowledge of particular fields, such as cleft surgery, is significantly improved by exposure to plastic surgery while in medical school. 20 Contrary to this study, plastic surgery was selected as the top specialty for treating cleft lip and cleft palate (selected by 90.9% of students) in a previous study evaluating perception of medical students on plastic surgery. 15 These findings contrast with those of Tanna et al, who found that oral and maxillofacial surgery was chosen by 78% of primary care residents, whereas only 57% connected treatment of this disorder with plastic surgeons. 21 Discrepancy in results can be attributed to different training patterns implemented by medical schools. It was observed that as the training stage increased, the likelihood of choosing plastic surgery for the treatment of cleft lip reduced. This observation might be explained by the fact that senior students who are doing a rotation in ENT consider cleft surgery as a part of only maxillofacial surgery. This underscores the fact that it is critical to educate students about the field of plastic surgery rather than relying on other specialties to do so. 15

Additionally, similar to the present study, data in a study 15 suggest that more than half of these students believed that hand surgeons were not plastic surgeons. This finding may be explained by several factors, such as the decreasing number of plastic surgeons performing hand surgery, the existence of an “orthopaedic” designated hand fellowship in several medical colleges, and limited training exposure, that is, only 30% of students received exposure to plastic surgery during their 4 years of training. 15 Plastic surgeons should broaden the formal instruction of medical students about hand and peripheral nerve surgery to improve their knowledge regarding these areas. The breadth of plastic surgery training within the NHS remains unclear, even at a higher level, especially concerning aesthetic surgery. 8 This study reveals that medical students still have a poor understanding of the spectrum of operations. According to earlier research, students were far more inclined to link cosmetic surgery with the field of plastic and reconstructive surgery. 22 This is probably impacted by how specialty is portrayed in the media. More than most surgical subspecialties, this one has a public profile. 23 Some authors have suggested changing the name since they believe the title's use of the word “plastic” contributes to the misconception. 24 These results are in-line with research done previously by Agarwal et al, who found that among U.S. medical students, hand surgery was the operation least associated with the specialty. 15

The present study findings are rather unsettling. This lack of comprehension has implications that go beyond the medical student. Previous research has demonstrated that beliefs formed during medical school carry over into general practice. 22 This affects the plastic surgery specialty in a variety of ways, such as lost referrals, deterring students from choosing plastic surgery as a specialization, losing the best prospects to other specialties, and losing the trust of the medical profession. Exposure to plastic surgery enhances a student's understanding of the field of study. Therefore, it is important to work to increase this exposure. Rotations in the clinical years would be the ideal way to accomplish this, but didactic lectures, career days, or workshops may also be included in the learning process during the preclinical years. 23

The present study has certain limitations. The design of a self-administered survey might not produce the best dependability and result in recollection bias. Being a single-center study, a nationwide study is required to provide a more generalizable and comprehensive result. A small sample size with a limited demographic data. Selection bias is a problem with voluntary online surveys because students interested in surgery are more likely to participate. Hence, it is possible that students who were more interested in surgery and, consequently, better educated about surgical subspecialties, were more likely to complete the survey. Additionally, because the results are from a single institution, local clinical and instructional experiences have an impact.

Despite these drawbacks, this study advances understanding of the matching procedure for a competitive specialty and adds knowledge of the factors that influence medical students' choice of a particular plastic surgery program. Academic residency programs that might want to modify their elective or interview formats would obtain meaningful insights from the present study. Future observational or cohort studies conducted over several years with a wider population of candidates for plastic surgery or different surgical specialties would be of great interest. Further research including numerous university medical institutions, especially those where plastic surgery is taught as a required course, will probably help us better understand how future doctors perceive plastic surgery. Additionally, distributing this survey to family doctors and the general public could help us gain a more comprehensive picture of current attitudes on plastic and reconstructive surgery.

### Conclusion

Medical students should be taught the broader scope of plastic surgery. In addition, inclusion of formal training during undergraduation is essence of time and should be added to or improved during plastic surgery rotations with more emphasis on the improvement areas of students' in plastic surgery (such as hand/peripheral nerve surgery). Teaching should concentrate on reconstructive and hand/peripheral nerve surgery. During medical school, student-led interest groups can be a useful tool for educating students about their specialty.
