# Supplementary material for: Medical Students Understanding of The Scope of Plastic Surgery
Source: Arch Plast Surg. 2024 Jan 24;51(2):251–7. doi: 10.1055/a-2219-2411 (PMC11001442; doi:10.1055/a-2219-2411)
Supplement: Supplementary file 1 — Supplementary Material [file 10-1055-a-2219-2411-s23mar0285oa.pdf]

Supplementary Material

Supplementary Table 1 This table depicts the clinical and preclinical medical students' perception towards the most suitable specialty to refer the stimulated cases

Criteria	Number (%)	General surgery	Ophthalmology	Orthopaedics	ENT	Plastic surgery	Neurosurgery	Vascular surgery	Urology	Maxillofacial	Other
Q1: A woman wants to reduce her breast size	Clinical	7 (77.8)	0	0	0	119 (50.6)	0	0	0	0	0
	Preclinical	2 (22.2)	0	0	0	116 (49.4)	0	0	0	0	0
	Total	9 (3.6)	0	0	0	235 (96.4)	0	0	0	0	0
Q2: A newborn child is diagnosed with cleft palate syndrome	Clinical	10 (76.9)	0	0	15 (78.9)	50 (69.4)	0	0	0	44 (43.6)	7 (35)
	Preclinical	3 (23.1)	0	8 (100)	4 (21.1)	22 (30.6)	6 (100)	5 (100)	0	57 (56.4)	13 (65)
	Total	13 (5.33)	0	8 (3.2)	19 (7.8)	72 (29.52)	6 (2.46)	5 (2.0)	0	101 (41.4)	20 (8.2)
Q3: An old woman diagnosed with carpal tunnel syndrome and has tingling in her fingers	Clinical	16 (72.7)	0	49 (68.1)	0	4 (26.7)	54 (54)	0	0	0	3 (14.3)
	Preclinical	6 (27.3)	11 (100)	23 (31.9)	0	11 (73.3)	46 (46)	3 (100)	0	0	18 (85.7)
	Total	22 (9.09)	11 (4.55)	72 (29.7)	0	15 (6.2)	100 (41.3)	1 (0.41)	0	0	21 (8.6)
Q4: Man suffers a fractured jaw	Clinical	0 (0)	0 (0)	38 (44.7)	13 (68.4)	0	0	0	0	74 (62.7)	1 (20)
	Preclinical	3 (100)	0 (0)	47 (55.3)	6 (31.6)	9 (100)	5 (100)	0	0	44 (37.3)	4 (80)
	Total	3 (1.23)	0 (0)	85 (34.8)	19 (7.7)	9 (3.69)	5 (2.0)	0	0	118 (48.36)	5 (2.0)
Q5: Man fell and broke his nose	Clinical	7 (38.9)	0 (0)	10 (50)	70 (69.3)	34 (47.2)	0	0	0	5 (20.8)	0
	Preclinical	11 (61.1)	0 (0)	10 (50)	31 (30.7)	38 (52.8)	9 (100)	0	0	19 (79.2)	0
	Total	18 (7.38)	0	20 (8.2)	101 (41.3)	72 (29.5)	9 (3.6)	0	0	24 (9.84)	0
Q6: Patient wants cosmetic nose reshaping	Clinical	0	0	0	18 (69.2)	108 (51.9)	0	0	0	0	0
	Preclinical	3 (100)	0	0	8 (30.8)	100 (48.1)	0	5 (100)	2 (100)	0	0
	Total	3 (1.22)	0	0	26 (10.66)	208 (85.2)	0	5 (2.05)	2 (0.82)	0	0
Q7: Child born with deformed skull affecting brain and growth	Clinical	8 (61.5)	0	11 (73.3)	0	6 (28.6)	88 (56.8)	0	0	3 (25)	10 (55.6)
	Preclinical	5 (38.5)	0	4 (26.7)	2 (100)	15 (71.4)	67 (43.2)	8 (100)	0	9 (75)	8 (44.4)
	Total	13 (5.33)	0	15 (6.1)	2 (0.8)	21 (8.61)	155 (63.5)	8 (3.28)	0	12 (4.91)	18 (7.3)
Q8: Patient working with a saw and cut his ulnar nerve	Clinical	4 (28.6)	0	29 (67.4)	0 (0)	8 (34.8)	84 (60)	1 (6.7)	0	0	0
	Preclinical	10 (71.4)	2 (100)	14 (32.6)	0	15 (65.2)	56 (40)	14 (93.3)	5 (100)	0	2 (100)

 © 2024. The Author(s).

Supplementary Table 1 (*Continued*)

Criteria	Number (%)	General surgery	Ophthalmology	Orthopaedics	ENT	Plastic surgery	Neurosurgery	Vascular surgery	Urology	Maxillofacial	Other
	Total	14 (5.74)	2 (0.82)	43 (17.62)	0	23 (9.43)	140 (57.3)	15 (6.15)	5 (2.04)	0	2 (0.82)
Q9: Patient has drooping eyelid, which affects his vision	Clinical	0 (0)	73 (48.7)	0	0	16 (100)	31 (63.3)	0	0	0	6 (46.2)
	Preclinical	0 (0)	77 (51.3)	0	16 (100)	0	18 (36.7)	0	0	0	7 (53.8)
	Total	0	150 (61.4)	0	16 (6.56)	16 (6.56)	49 (20.)	0	0	0	13 (5.3)
Q10: Man has exposed tibia after an accident. A muscle coverage from his back could save the leg.	Clinical	14 (50)	0	67 (50.8)	0	41 (68.3)	0	4 (22.2)	0	0	0
	Preclinical	14 (50)	0	65 (49.2)	0	19 (31.7)	0	14 (77.8)	0	0	6 (100)
	Total	28 (11.4)	0	132 (54.1)	0	60 (24.59)	0	18 (7.38)	0	0	6 (2.45)
Q11: Patient has nasal septal deviation and breathing problems	Clinical	3 (33.3)	0	0	110 (56.1)	7 (33.3)	0	0	0	6 (33.3)	0
	Preclinical	6 (66.7)	0	0	86 (43.9)	14 (66.7)	0	0	0	12 (66.7)	0
	Total	9 (3.69)	0	0	196 (80.3)	21 (8.61)	0	0	0	18 (7.37)	0
Q12: An old woman has to undergo total hip replacement	Clinical	0 (0)	0	123 (58)	0	2 (18.2)	0	1 (100)	0	0	0
	Preclinical	11 (100)	2 (100)	89 (42)	2(10)	9 (81.8)	0	0	5 (100)	0	0
	Total	11 (4.51)	2 (0.82	212 (86.8)	2 (0.8)	11 (4.51)	0	1 (0.4)	5 (2.05)	0	0
Q13: Patient has lacerated hand tendon	Clinical	15 (50)	0	82 (60.7)	0	11 (50)	4 (25)	7 (58.3)	0	0	7 (38.9)
	Preclinical	15 (50)	0 (0)	53 (39.3)	4 (100)	11 (50)	12 (75)	5 (41.7)	5 (100)	2 (100)	11 (61.1)
	Total	30 (12.3)	0	135 (55.33)	4 (1.64)	22 (9.02)	16 (6.5)	12 (4.91)	5 (2.05)	2 (0.81)	18 (7.3)
Q14: Infant born without an outer ear	Clinical	3 (33.3)	0	0	70 (55.6)	42 (63.6)	5 (71.4)	0	0	3 (42.9)	3 (15)
	Preclinical	6 (66.7)	2 (100)	2 (100)	56 (44.4)	24 (36.4)	2 (28.6)	5 (100)	0	4 (57.1)	17 (85)
	Total	9 (3.69)	2 (0.82)	2 (0.8)	126 (51.6)	66 (27.05)	7 (2.8)	5 (2.05)	0	7 (2.87)	20 (8.1)
Q15: Cosmetic contouring and suction of fat (liposuction)	Clinical	14 (77.8)	0	0	0	112 (54.1)	0	0	0	0	0
	Preclinical	4 (22.2)	0	9 (100)	5 (100)	95 (45.9)	0	0	0	3 (100)	2 (100)
	Total	18 (7.38)	0	9 (3.6)	5 (2.05)	207 (84.8)	0	0	0	3 (1.23)	2 (0.83)
Q16: Woman with repeatedly infected permanent filler in face	Clinical	12 (57.1)	0	0	4 (50)	82 (51.2)	0	0	4 (100)	24 (72.7)	0
	Preclinical	9 (42.9)	5 (100)	0	4 (50)	78 (48.8)	4 (100)	4 (100)	0	9 (27.3)	5 (100)
	Total	21 (8.61)	5 (2.0)	0	8 (3.28)	160 (65.57)	4 (1.6)	4 (1.64)	4 (1.64)	33 (13.5)	5 (2.0)

(*Continued*)

 © 2024. The Author(s).

Supplementary Table 1 (*Continued*)

Criteria	Number (%)	General surgery	Ophthalmology	Orthopaedics	ENT	Plastic surgery	Neurosurgery	Vascular surgery	Urology	Maxillofacial	Other
Q17: Excision of a lipoma in back	Clinical	104 (55.6)	0	0	0	16 (66.7)	4 (100)	2 (33.3)	0	0	0
	Preclinical	83 (44.4)	2 (100)	0	0	8 (33.3)	0	4 (66.7)	0	0	19 (100)
	Total	187 (77.2)	2 (0.83)	0	0	24 (9.9)	4 (1.6)	6 (2.48)	0	0	19 (7.8)
Q18: Fireman suffers from abdominal chest burn	Clinical	28 (62.2)	0	0	0	82 (58.2)	0	5 (31.3)	0	0	11 (45.8)
	Preclinical	17 (37.8)	3 (100)	2 (100)	5 (100)	59 (41.8)	2 (100)	11 (68.8)	0	4 (0)	13 (54.2)
	Total	45 (18.6)	3 (1.24)	2 (0.83)	5 (2.0)	141 (58.2)	2 (0.8)	16 (6.61)	0	4 (1.65)	24 (9.9)
Q19: Motorcycle accident Patient has brachial plexus injury	Clinical	7 (58.3)	0	48 (71.)	0	5 (50)	62 (52.5)	3 (30)	0	1 (20)	0
	Preclinical	5 (41.7)	5 (100)	19 (28.4)	7 (100)	5 (50)	56 (47.5)	7 (70)	0	4 (80)	8 (100)
	Total	12 (4.96)	5 (2.07)	67 (27.6)	7 (2.8)	10 (4.13)	118 (48.7)	10 (4.13)	0	5 (2.07)	8 (3.3)
Q20: Patient with protrusion of the eyes for decompression surgery	Clinical	2 (100)	95 (53.1)	1 (100)	0	8 (0)	13 (65)	2 (100)	0	5 (55.6)	0
	Preclinical	0 (0)	84 (46.9)	0	4 (100)	3 (27.3)	7 (35)	0	5 (100)	4 (44.4)	9 (100)
	Total	2 (0.83)	179 (73.9)	1 (0.4)	4 (1.6)	11 (4.55)	20 (8.2)	2 (0.83)	5 (2.0)	9 (3.72)	9 (3.7)
Q21: Patient has repeated vomiting and CT scan shows bowel obstruction	Clinical	121 (60.2)	0	0	0	0	0	0	5 (25)	0	0
	Preclinical	80 (39.8)	0	0	7 (100)	5 (100)	2 (100)	2 (100)	15 (75)	0	5 (100)
	Total	201 (83)	0	0	7 (2.89)	5 (2.07)	2 (0.83)	2 (0.83)	20 (8.25)	0	5 (2.0)
Q22: Heavy smoker diagnosed with cancer in roof of the mouth	Clinical	15 (51.7)	0	0	35 (49.3)	0	0	0	0	62 (59)	14 (56)
	Preclinical	14 (48.3)	2 (100)	0	36 (50.7)	5 (100)	5 (5)	0	0	43 (41)	11 (44)
	Total	29 (11.9)	2 (0.83)	0	71 (29.3)	5 (2.07)	5 (2.0)	0	0	105 (43.3)	25 (10.3)
Q23: Patient stabbed with a nail in the lower lid and he needs orbital fracture repair	Clinical	2 (8)	71 (57.7)	12 (50)	0	11 (84.6)	0	0	0	30 (73.2)	0
	Preclinical	23 (92)	52 (42.3)	12 (50)	3 (100)	2 (15.4)	7 (100)	0	0	11 (26.8)	6 (100)
	Total	25 (10.3)	123 (50.8)	24 (9.9)	3 (1.2)	13 (5.37)	7 (2.8)	0	0	41 (16.94)	6 (2.48)

 © 2024. The Author(s).
